# Influence of Wearing Surgical Mask on Interpersonal Space Perception Between Mainland Chinese and Taiwanese People

**DOI:** 10.3389/fpsyg.2021.692404

**Published:** 2021-09-03

**Authors:** Yu-Chi Lee, Yi-Lang Chen

**Affiliations:** ^1^School of Design, South China University of Technology, Guangzhou, China; ^2^Department of Industrial Engineering and Management, Ming Chi University of Technology, New Taipei, Taiwan

**Keywords:** COVID-19, interpersonal space, face mask, social distancing, proximity

## Abstract

Wearing face masks and maintaining social distancing of 1.5m are two common preventive measures against the spread of COVID-19. However, the interaction of these preventive measures in interpersonal space (IPS) perception remains unknown. This study evaluated the effects of wearing surgical masks, sex dyads, and approaching patterns on IPS judgment. Data were collected from participants from Mainland China (*n*=100) and Taiwan (*n*=100) through an online survey. Therefore, the regional differences were also examined. A smaller IPS was observed when participants faced confederates wearing surgical masks than in the no-mask condition. Female dyads tended to maintain a smaller IPS than did both male and mixed-sex dyads, and Taiwanese participants maintained a significantly larger IPS than did Mainland Chinese participants. No significant difference was observed between the active and passive pattern. Moreover, the interaction between region and mask had a significant influence on IPS perception. Among all test combinations, only the IPS perceived by Taiwanese participants facing confederates without surgical masks exceeded 1.5m. This study revealed that the wearing of surgical masks for health protection during the pandemic influences IPS perception in different regions. The current findings may provide useful information for social interaction and environmental design during the COVID-19 pandemic.

## Introduction

As of the beginning of 2021, more than 100 million confirmed COVID-19 cases have been recorded worldwide. To combat the pandemic, the WHO recommends several methods to prevent the spread of COVID-19, including washing hands frequently and correctly, maintaining social distancing of approximately 1.5m and 1.0m indoors and outdoors, respectively, and wearing a face mask when going out and staying indoor environments (Khosronejad et al., 2020). Among these preventive measures, maintaining a distance of 1.5m from others has been mandated in many public areas. This rule was imposed because respiratory viruses, such as coronaviruses and influenza, infect people through droplet inhalation (Sajed and Amgain, 2020) and aerosol transmissions (Santarpia et al., 2020; Tang et al., 2020).

With regard to social distancing, Chu et al. (2020) conducted a meta-analysis and reported that maintaining a larger distance (>1m) could effectively improve self-protection and reduce the risk of infection. Another recommended measure for self-protection is to wear a surgical mask (Johnson et al., 2009). Studies that have assessed the effect of face mask wearing on the spread of COVID-19 in the clinical and medical fields have consistently found that using face masks is an effective method to contain the spread of the virus (Bae et al., 2020; Matuschek et al., 2020). Chu et al. (2020) reported that wearing a face mask, especially N95 masks, can significantly reduce the risk of infection. Although the aforementioned preventive measures seem to be effective, their implementation has resulted in changes to people’s lifestyles and habits. People’s facial expressions are covered when they use a face mask, which in turn, affects their feelings and cognitions during social interaction (Cartaud et al., 2020a). In the context of widespread COVID-19 infections, people have become accustomed to wearing face masks and maintaining a 1.5-m social distance in daily life. The changed behaviors led to discomfort, heightened arousal, and limited social signaling (Welsch et al., 2020), as well as affected to the interpersonal space (IPS) perception (Cartaud et al., 2020a; Coello and Cartaud, 2021; Iachini et al., 2021).

The concept of IPS, which was first proposed by Hall (1966), refers to the limit of comfortable distance between people and has been widely studied in the field of psychology, environmental design, and human–machine interaction. IPS perception is mainly affected by visual cues. Factors influencing IPS can be roughly classified into three categories, namely, participant characteristics, confederate features, and environmental conditions. Regarding participant characteristics, studies have indicated that women maintain a greater IPS than males do (Remland et al., 1995; Uzzell and Horne, 2006; Iachini et al., 2016; Yu et al., 2020). Furthermore, participants’ feelings (Iachini et al., 2015) and type of gaze (Ioannou et al., 2014; Sicorello et al., 2019) affect IPS measurements. The IPS maintained by participants with autism spectrum disorders or restrictive-type anorexia has also been studied (Gessaroli et al., 2013; Nandrino et al., 2017) and used for further treatment and prediction. Confederate features, such as sex (Uzzell and Horne, 2006; Ruggiero et al., 2019), facial expression (Cartaud et al., 2018), height (D’Angelo et al., 2019), age (Iachini et al., 2016), body shape (Bailenson et al., 2003), and occupation (Aliakbari et al., 2011), have also been investigated. Moreover, research has examined environmental factors, such as approaching direction (Hecht et al., 2019; Yu et al., 2020; Candini et al., 2021) and pattern (Iachini et al., 2014; Yu et al., 2020), music type (Tajadura-Jiménez et al., 2011), temperature (Ruggiero et al., 2019), virtual environment (Iachini et al., 2014, 2016), and culture (Remland et al., 1995). The results of these studies have contributed substantially to clarifying the effects of human psychology, interaction, and environmental factors on IPS. Coello and Cartaud (2021) also proposed a theory to express the relationship between the degree of threat and IPS based on psychophysiological evidence. When people feel threatened or insecure, they maintain a larger IPS as a self-protection mechanism. When a face mask is worn, the confederate presents a risky, dangerous, and negative impression (Abney, 2018) that leads to psychological barriers (Burgess and Horii, 2012). Because people are expected to comply with WHO guidelines regarding the wearing of surgical masks during the COVID-19 pandemic, atypical behaviors may influence IPS. Research on the impact of wearing surgical masks on perceptual IPS is scarce.

Cartaud et al. (2020b) first employed online interviews to evaluate the effects of wearing face masks and the consequent facial expressions on the IPS of 457 French participants; the results showed that IPS was significantly reduced when confederates were wearing a face mask, as the participants were perceived as more trustworthy compared to the other conditions. Calbi et al. (2021) conducted an online survey on face covering on a sample of 96 Italians and found that people react to various types of protective face covering in different manners. Generally, cultural norms are a crucial factor affecting IPS (Hall, 1966), and contact cultures typically exhibit a smaller IPS than do noncontact culture (Hall, 1966; Baldassare and Feller, 1975). IPS perceptions also differ by ethnicity (Beaulieu, 2004; Sorokowska et al., 2017; Sicorello et al., 2019). People who live in Mainland China and Taiwan have the same ethnic background and belong to noncontact cultures. The lifestyle, risk cognition, and culture for both groups still differ. This motivated us to examine the regional differences in the behavioral changes (IPS in the study) under the threatening environments, particularly the pandemic first outbreak in Mainland China.

Even though factors influencing IPS have been identified, uncertainty remains regarding the impact of wearing surgical masks on IPS judgment. Therefore, this study examined the effects of region, surgical mask wearing, sex dyads, and approaching pattern on IPS perception. Understanding the changes in IPS on the basis of these variables may provide useful information on human interaction during the COVID-19 pandemic.

## Materials and Methods

### Participants

Two hundred participants were recruited in the study. Among them, 100 participants were Mainland Chinese (50 women), and their age and height were 23.38±1.47years and 175.30±3.76cm for male participants and 22.42±1.73years and 162.82±5.39cm for female participants. The remaining 100 male and female participants (50 each) were Taiwanese, and their age and height were 22.96±2.13years and 174.10±6.50cm for male participants and 22.44±1.63years and 161.30±5.42cm for female participants. To evaluate the differences in age and height between Mainland Chinese and Taiwanese participants, the independent *t*-test was applied. The results showed that no significant differences in age (*t*=0.790, degree of freedom=198, *p*=0.430) and height (*t*=1.160, degree of freedom=198, *p*=0.248) between the two groups were found. The data were collected from June to November, 2020. All participants reported normal vision and no cognitive or mental problems. Furthermore, all participants were right-handed and not familiar with the confederates in the experiment. Participants were fully informed of the testing procedure and were asked to sign a consent form before the data collection. The South China University of Technology Institutional Ethics Committee approved the experimental procedures. These two groups were selected for comparison because COVID-19 first appeared in Mainland China and the pandemic situation in Taiwan was relatively stable in terms of epidemic control.

### Experimental Setting

**Figure 1:**
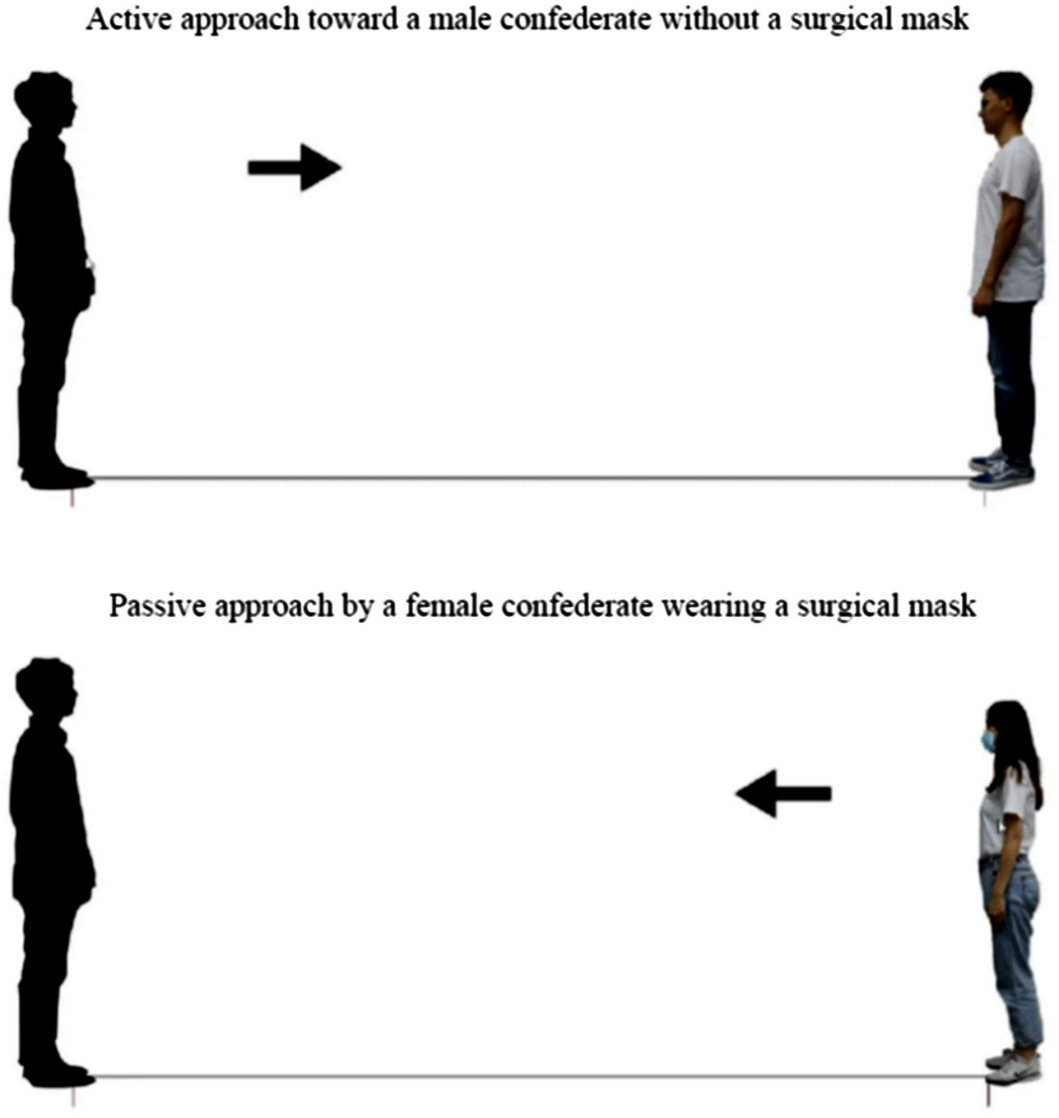
Screenshots of the online survey under various conditions.

Because of the COVID-19 pandemic, the study was forced to use an online survey to collect data on IPS to avoid human-to-human transmission (Ruggiero et al., 2019; Calbi et al., 2021). The online survey was adapted from the paper-and-pencil test utilized in the research of Hayduk (1983) and Xiong et al. (2020). An online survey is an effective method for collecting data on IPS in different countries and is widely used in clinical and practical investigations (Iachini et al., 2016). The paper-and-pencil test was regarded as a projective measurement in which participants were instructed to imagine that they were the people on the paper, as illustrated in Figure 1. A computer with the Axure RP rapid prototyping tool (Axure Software Solutions, Inc., San Diego, CA, United States) was used to conduct the survey. Moreover, an experimenter used video conference system and remote desktop connection software to execute the online test. All the materials were shown on the desktop of the experimenter’s computer. Each participant was asked to use the remote desktop connection to control the experimenter’s computer. The video conference system was used for communication, and the remote desktop connection software was applied for monitoring. The above two technologies were employed to guide the participants one-by-one to minimize the testing bias. During the test, participants were instructed to use the mouse cursor to move the virtual subject (avatar) to approach the confederate in the active pattern and to move the digital confederate to approach the digital participant in the passive pattern (Figure 1). When a participant started to move the avatar, the arrow guiding the movement direction between the two avatars was hidden to avoid affecting the participant’s distance judgment. That is, no reference was provided for the distance between the two avatars during the IPS determination, except for the changes in spatial perception caused by moving the avatar. The participants were asked to imagine and then determine the IPS by moving the avatar to a position that still felt comfortable but had just started to feel uncomfortable. The IPS was defined as in previous studies (Hayduk, 1983; Adams and Zuckerman, 1991; Nandrino et al., 2017; Yu et al., 2020). Subsequently, the distance between the two avatars was transformed at a 1:30 ratio to obtain the psychological interpersonal distance (Sorokowska et al., 2017). The distance between the two avatars was originally set at 13.33cm, which means that the initial distance between the participant and the confederate was approximately 4m in the real world (Yu et al., 2020). Prior to the test, the experimenter confirmed the experimental settings (e.g., voice quality, mouse cursor controls, and displays) with each participant because the remote desktop connection software was applied in the test. The reliability of the measurement used in the test was examined through a pilot study. The intraclass correlation coefficients (ICC) estimates and their 95% confident intervals were calculated using SPSS statistical package version 23 (SPSS Inc., Chicago, IL, United States) based on a single-measure, absolute-agreement, and 2-way mixed-effects model ICC (3,1) as indicated by Koo and Li (2016). The results reported that the ICCs between the repetitions were 0.85 and 0.79 in the active and passive patterns, respectively. According to the standard proposed by Koo and Li (2016), an ICC value ranging from 0.75 to 0.90 indicates good reliability. Thus, the measurement used in the study had satisfactory reliability.

### Confederates

A man and a woman aged 23years with typical Chinese appearance were selected as confederates. The heights of the male and female confederates were 174.3 and 161.7cm, respectively. The confederates were dressed in casual clothing (jeans and a white t-shirt) without any accessories. A digital camera (Sony HDR-XR260; Sony Corporation, Minato, Tokyo, Japan) was used to capture the sagittal view of the two confederates under two conditions: with and without surgical mask wearing. The sagittal images were served as digital confederates in the online survey. The confederates were requested to maintain a neutral expression during the image capture and were not known to the participants. The heights of the digital male and female confederates on the screen were scaled down to 58.1mm and 53.9mm, respectively. The surgical mask used in the test was blue and without any decoration, which represents the type of face mask commonly recommended during the COVID-19 pandemic.

### Procedure

Before the test, the experimenter explained the survey procedure to the participants. A 2-min video produced by Stanford Medicine was used to introduce the COVID-19 pandemic to the participants to enable them to recall how they felt during the pandemic. Furthermore, the sagittal view of the confederates with and without a surgical mask was displayed for the corresponding condition when the participants made the perceptual distance judgment. The images were used to help the participants imagine the feeling of facing the confederates under various situations to ensure IPS data quality. Each participant was requested to complete three repetitions, and the average value of the trials was calculated for the analysis. A minimum 3-min rest period was provided to each participant between the trials. The trials were presented one-by-one for IPS judgment and were randomly arranged. For the IPS judgment, the participants used the mouse to move the avatar to a position where they psychologically felt close to uncomfortable but still comfortable. Each participant had the opportunity to make slight adjustments to the position of their avatar to confirm the perceived distance. Once the participant determined the IPS, the computer automatically calculated and recorded the distance between the chins of the two avatars (Yu et al., 2020). The same procedures and materials were used to perform the tests on Mainland Chinese and Taiwanese participants. As a result, 1,600 data samples (200 participants × 2 confederates × 2 mask conditions × 2 approach patterns) were collected in the study for subsequent analysis.

### Statistical Analysis

The independent variables in the study were region (Mainland Chinese or Taiwanese), sex dyads (male–male, mixed sex, and female–female), face mask (with or without), and approach patterns (active or passive). The dependent variable was the IPS distance in centimeters. Data were analyzed using SPSS 23.0 (SPSS Inc., Chicago, IL, United States) and the significance level (α) was set at 0.05. Four-way ANOVA was conducted to evaluate the effect of independent variables, and the Scheffé method was used for post-hoc comparisons. The effect size was determined using *η*^2^ value for each significant effect. Beforehand, the Kolomogorov-Smimov test was used to verify the compliance of numerical variables with the normal distribution, while the Levene’s test was used to verify the homogeneity of variances.

## Results

**Figure 2:**
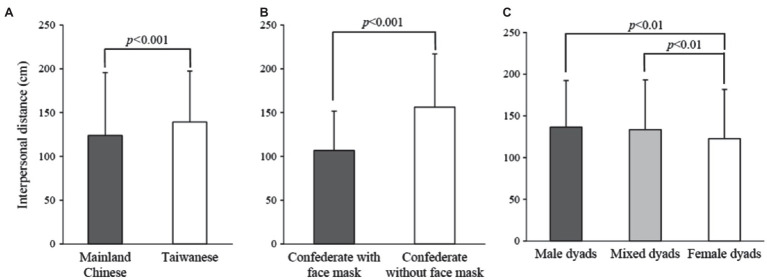
Mean interpersonal distance with the standard deviations under each main effect including **(A)** region, **(B)** face mask, and **(C)** sex dyads compared using the Scheffé’s method.

Through Kolmogorov–Smirnov test, the IPS data collected in the study were normally distributed (*D*_(1600)_=0.009, *p*=0.052) meanwhile Levene’s test showed the data were homogenous (*F*_(23,1,576)_=1.238, *p*=0.201). Table 1 presents the four-way ANOVA results of the IPS measurements. Region (*F*_(1,1,576)_=27.210, *p*<0.001, *η*^2^=0.017), sex dyads (*F*_(2,1,576)_=8.126, *p*<0.001, *η*^2^=0.010), and face mask wearing (*F*_(1,1,576)_=316.483, *p*<0.001, *η*^2^=0.167) significantly influenced the IPS, whereas the approach variable (active or passive pattern) did not result in a difference in IPS. The Taiwanese participants exhibited a significantly larger IPS than did the Mainland Chinese participants (Figure 2A). The Taiwanese and Mainland Chinese IPS were 139.24±58.28cm and 123.95±71.86cm, respectively. The difference in IPS between the two regions was 15.25±2.9cm (95%CI: 9.53–20.97, *p*<0.001) for both sexes. Participants maintained a significantly longer distance when facing a confederate who was not wearing a surgical mask (156.32±60.7cm) than when the surgical mask was worn (106.83±44.79cm), with a difference of 49.49±2.67cm (95%CI: 44.26–54.72, *p*<0.001; Figure 2B). For the post-hoc test results, female dyads maintained a significantly smaller IPS (122.67±59.17cm) than the male dyads (136.64±55.65cm, with a difference of 13.97±3.72cm, 95%CI: 4.86–23.07, *p*<0.01) and the mixed dyads (133.50±59.71cm, with a difference of 10.82±3.22cm, 95%CI: 2.94–18.71, *p*<0.01; Figure 2C). No significant difference in IPS was observed between the male dyads and the mixed dyads.

**Table 1:** Four-way ANOVA results on interpersonal distance.

Variables	Degrees of freedom	*F-*value	Significance	*η* ^2^
Region	1	27.210	*p* <0.001	0.017
Sex dyads	2	8.126	*p* <0.001	0.010
Face mask	1	316.483	*p* <0.001	0.167
Approach	1	0.103	*p* =0.748	–
Region × Sex dyads	2	2.265	*p* =0.104	–
Region × Face mask	1	5.892	*p* <0.05	0.004
Region × Approach	1	1.812	*p* =0.178	–
Sex dyads × Face mask	2	0.720	*p* =0.487	–
Sex dyads × Approach	2	0.311	*p* =0.733	–
Face mask × Approach	1	0.001	*p* =0.980	–
Region × Sex dyads × Face mask	2	1.031	*p* =0.357	–
Region × Sex dyads × Approach	2	0.230	*p* =0.794	–
Region × Face mask × Approach	1	0.081	*p* =0.776	–
Sex dyads × Face mask × Approach	2	0.484	*p* =0.616	–
Region × Sex dyads × Face mask × Approach	2	0.219	*p* =0.803	–
Error	1576			

**Figure 3:**
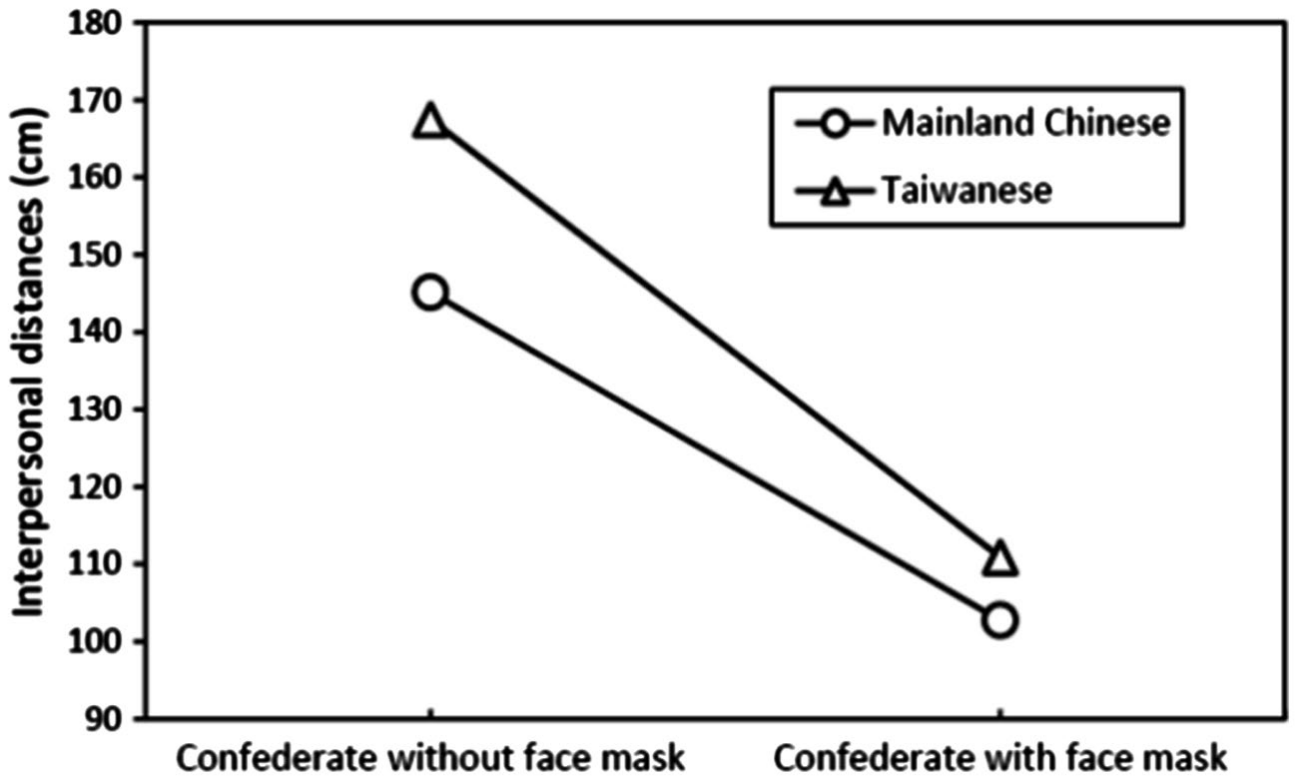
Interaction effect of region and face mask on interpersonal space perceptions.

No significant interaction effect was observed, except for region × face mask (*F*_(1, 1,576)_=5.892, *p*<0.05, *η*^2^=0.004; Table 1). The largest IPS was noted in the condition of Taiwanese participants facing confederates without a surgical mask (167.50±57.17cm), followed by Mainland Chinese participants facing confederates without a surgical mask (145.13±62.11cm), Taiwanese participants facing confederates with a surgical mask (110.90±44.05cm), and Mainland Chinese participants facing confederates with a surgical mask (102.76±45.20cm; Figure 3). No significant difference was observed between the two groups when facing confederates wearing a surgical mask.

## Discussion

During the COVID-19 pandemic, in addition to frequent hand washing, wearing face masks and maintaining social distance (1.5m) have become the two main self-protection methods; these measures were suggested from the perspectives of physical and individual hygiene. Even though the spread of COVID-19 has slowed with the development of vaccines, these preventive measures may continue to change people’s lifestyle and work. IPS plays a vital role in influencing the behaviors pertaining to human communication and interaction. Although people’s lifestyles and working conditions have changed, comprehensive information on IPS remains scarce. Therefore, this study conducted online surveys to evaluate the effects of factors influencing IPS perception during the COVID-19 pandemic.

In the research of Hall (1966) and Baldassare and Feller (1975), cultural norms (contact and noncontact) are a crucial factor affecting IPS. Moreover, significant differences have been observed in IPS perception among regions and ethnicities (Beaulieu, 2004; Sorokowska et al., 2017; Sicorello et al., 2019). Although people in Mainland China and Taiwan have the same ethnic background and belong to noncontact cultures, this study found that the Taiwanese participants preferred maintaining a significantly larger IPS than did the Mainland Chinese participants. On average, the difference in IPS observed between these two regional participant groups was approximately 15cm. Yu et al. (2020) compared the IPS maintained by ethnic Chinese people with that maintained by other ethnicities and reported that external environmental factors and lifestyle are potential factors affecting IPS. Compared with Taiwanese participants, the Mainland Chinese participants were more accustomed to crowds because of the large number of people in some public areas. Therefore, they were used to maintaining a shorter distance from strangers in public (e.g., on busses, in queues, and on the subway). This is one of the possible reasons for the difference in IPS between these two groups. However, Welsch et al. (2020) indicated that people have tended to maintain a larger IPS during the COVID-19 pandemic. This pandemic first appeared in Mainland China in December 2019, and after a few months, it gradually subsided in both Mainland China and Taiwan. The data collection period of this study was June–November 2020. The current results highlight a considerable region effect on IPS (i.e., Taiwanese IPS > Mainland Chinese IPS), indicating that the Taiwanese participants tended to be more conservative in terms of IPS during the COVID-19 pandemic than the Mainland Chinese participants. IPS data from before and during the COVID-19 pandemic should be further compared in order to obtain a more comprehensive understanding of the changes in human behaviors.

Previous studies have investigated the effect of sex dyads on IPS but no consistent results have been obtained. Consistent with previous research (Caplan and Goldman, 1981; Aliakbari et al., 2011), Yu et al. (2020) reported that male dyads maintained the largest IPS and female dyads maintained the smallest one. By contrast, Baxter (1970) and Evans and Howard (1973) revealed that the smallest IPS was observed in mixed-sex dyads. Furthermore, Hecht et al. (2019) found that the IPS maintained by mixed-sex dyads was no significant difference to that maintained by same-sex dyads. Zhou et al. (2019) reported that the social interaction distance between people was related to perceptual judgments concerning social grouping; Chinese participants tended to maintain a longer distance in mixed-sex dyads because they felt insecure and shy (Yang, 1988). The results of the current study are in line with related findings that participants maintained greater distance when they are in mixed-sex dyads and that the shortest distance was maintained in female dyads.

When people feel threatened, such as facing the confederates with angry or invisible facial expressions, they subconsciously adjust their IPS to feel psychologically comfortable (Ruggiero et al., 2017, 2021; Cartaud et al., 2018, 2020a). Feeling comfortable and safe by increasing IPS are a psychological adjustment mechanism (Coello and Cartaud, 2021). For example, a larger IPS was reported when participants faced confederates with a gaze (Sicorello et al., 2019), negative expressions (Iachini et al., 2015), aggressive emotions (Tajadura-Jiménez et al., 2011), tall stature (D’Angelo et al., 2019), and large body size (Bailenson et al., 2003). The social impression cast by face masks was investigated by Abney (2018) and Burgess and Horii (2012). Their results indicated that people wearing face masks generated feelings of risk, danger, and harm; that is, negative emotions may cause people to maintain a greater IPS. Among relevant investigations, Cartaud et al. (2020b), employing a questionnaire, were the first to report that wearing a face mask as a preventive measure against COVID-19 resulted in a reduction of IPS distance among French participants. Similarly, the present study results indicate that confederates with or without a surgical mask significantly influenced IPS judgments, regardless of the region variable. Notably, participants facing the confederate without a surgical mask reported a significantly larger IPS, with an increment of 50% (with/without surgical mask: 106.83cm/156.32cm). Most participants in this study reported that they experienced a strong sense of aggression and insecurity when facing the confederate without a surgical mask, which resulted in a larger IPS. The results could be attributed to the disease-avoidance mechanisms. There is a unique system composed of various cognitive and affective processes and behaviors with the main goal of protecting the organism from coming into contact with the infectious disease in the first place. Such as the behavioral immune system, as defined by Schaller (2006), plays a unique role in shaping a variety of human behaviors, from basic avoidance of rotten food to social cognitions. By contrast, participants tended to maintain a shorter social distance from the confederate wearing a surgical mask. This means that the use of surgical masks may provide a sense of safety to people (Cartaud et al., 2020a) due to the emotion of the confederate might be less detected (Carbon, 2020). Notably, the psychological feelings and impressions generated by the wearing of a face mask during the COVID-19 pandemic are the exact opposite of past experience. These findings imply that the external environment, social atmosphere, and health knowledge may induce changes in humans’ perception of specific products, in turn altering human behavior.

Iachini et al. (2014, 2016) conducted simulations in a virtual reality environment and reported that the comfortable distance in the passive pattern was larger than that in the active pattern. In the present study, however, no significant difference in IPS was noted between the patterns. The use of different measurement methods may have caused the inconsistent results. In the passive pattern, participants were generally requested to say “stop” to make the confederate stand at the point at which the participants started to feel uncomfortable. The participants were immobile and passive when determining their IPS in a given situation. A feeling of insecurity and pressure was thus induced, which affected IPS perceptions (Ruggiero et al., 2017). In our study, avatars were used to represent the participants according to their sex, and sagittal view images of real confederates were used for evaluation (Figure 1). Participants were asked to use their imagination to determine their IPS when moving the avatar toward the confederate’s image in the active pattern and when moving the confederate’s image toward the avatar in the passive pattern. The use of a questionnaire to collect data on IPS has been widely employed in related investigations (Hayduk, 1983; Iachini et al., 2016; Xiong et al., 2020) and our measurement was reliable (ICC=0.82); thus, the absence of visual cues, body somatosensory information, and interactivity may explain the inconsistent results. Nevertheless, the current findings are consistent with those of Hecht et al. (2019), who indicated that the approach patterns had no effect on IPS perception when participants were facing a simplified avatar. Notably, diverse measurement methods may produce different results when determining the psychologically comfortable distance. To compare the present study’s IPS results with those of previous studies, the measurement of IPS should be considered. Clarifying the differences among the measurements of IPS judgment is thus essential.

This study has some limitations. Because of the pandemic, an online survey was employed for the evaluation. The IPS data obtained under this situation may thus be inconsistent with those obtained in real-world measurement. In addition, only blue surgical masks were used in the study. The effects of face masks of various types and colors on IPS perception should be further investigated to provide more information pertaining to social distancing assessment, social interaction enhancement, and environmental design improvement.

## Conclusion

The effects of region, presence or absence of a face mask, sex dyads, and approach pattern on IPS were evaluated in this study. All main effects had a significant influence on IPS determination, except the approach pattern. The Taiwanese participants required a greater distance for psychological comfort than the Mainland Chinese participants did. When facing a confederate who did not wear a face mask, the participants tended to maintain a larger IPS. Female dyads maintained a shorter distance than did male and mixed-sex dyads. The results of the interaction of region and face mask demonstrated that Taiwanese participants maintained the longest distance from a confederate without a face mask, whereas the Mainland Chinese participants maintained the shortest distance when encountering a masked confederate. People’s lifestyles and habits have undeniably been affected by the COVID-19 pandemic. Changes in behavior may affect people’s performance pertaining to psychological, physiological, and social interaction. More in-depth and extensive investigations will be practically valuable for clarifying the differences in and the application of human behaviors during the COVID-19 pandemic.

## Data Availability Statement

The original contributions presented in the study are included in the article/supplementary material, further inquiries can be directed to the corresponding author.

## Ethics Statement

The studies involving human participants were reviewed and approved by the South China University of Technology Institutional Ethics Committee (May 1, 2020). Participants provided written informed consent to participate in this study. Written informed consent was obtained from the individual(s) for the publication of any potentially identifiable images or data included in this article.

## Author Contributions

All authors listed have made substantial, direct, and intellectual contributions to this research and approved it for publication.

## Conflict of Interest

The authors declare that the research was conducted in the absence of any commercial or financial relationships that could be construed as a potential conflict of interest.

## Publisher’s Note

All claims expressed in this article are solely those of the authors and do not necessarily represent those of their affiliated organizations, or those of the publisher, the editors and the reviewers. Any product that may be evaluated in this article, or claim that may be made by its manufacturer, is not guaranteed or endorsed by the publisher.

## References

[ref1] Abney K. (2018). “Containing” tuberculosis, perpetuating stigma: the materiality of N95 respirator masks. Anthropol. South Afr. 41, 270–283. 10.1080/23323256.2018.1507675

[ref2] Adams L. Zuckerman D. (1991). The effect of lighting conditions on personal space requirements. J. Gen. Psychol. 118, 335–340. 10.1080/00221309.1991.9917794

[ref3] Aliakbari M. Faraji E. Pourshakibaee P. (2011). Investigation of the proxemic behavior of Iranian professors and university students: effects of gender and status. J. Pragmat. 43, 1392–1402. 10.1016/j.pragma.2010.10.021

[ref4] Bae S. Kim M. C. Kim J. Y. Cha H. H. Lim J. S. Jung J.. (2020). Effectiveness of surgical and cotton masks in blocking SARS–CoV-2: A controlled comparison in 4 patients. Ann. Intern. Med. 173, W22–W23. 10.7326/m20-1342, PMID: 32251511 PMC7153751

[ref5] Bailenson J. N. Blascovich J. Beall A. C. Loomis J. M. (2003). Interpersonal distance in immersive virtual environments. Personal. Soc. Psychol. Bull. 29, 819–833. 10.1177/0146167203029007002, PMID: 15018671

[ref6] Baldassare M. Feller S. (1975). Cultural variations in personal space: theory, methods, and evidence. Ethos 3, 481–503. 10.1525/eth.1975.3.4.02a00020

[ref7] Baxter J. C. (1970). Interpersonal spacing in natural settings. Sociometry 33, 444–456. 10.2307/2786318, PMID: 5483948

[ref8] Beaulieu C. (2004). Intercultural study of personal space: A case study. J. Appl. Soc. Psychol. 34, 794–805. 10.1111/j.1559-1816.2004.tb02571.x

[ref9] Burgess A. Horii M. (2012). Risk, ritual and health responsibilisation: Japan’s ‘safety blanket’ of surgical face mask-wearing. Sociol. Health Illn. 34, 1184–1198. 10.1111/j.1467-9566.2012.01466.x, PMID: 22443378

[ref10] Calbi M. Langiulli N. Ferroni F. Montalti M. Kolesnikov A. Gallese V.. (2021). The consequences of COVID-19 on social interactions: an online study on face covering. Sci. Rep. 11, 1–10. 10.1038/s41598-021-81780-w, PMID: 33510195 PMC7844002

[ref11] Candini M. Battaglia S. Benassi M. di Pellegrino G. Frassinetti F. (2021). The physiological correlates of interpersonal space. Sci. Rep. 11:2611. 10.1038/s41598-021-82223-2, PMID: 33510396 PMC7844010

[ref12] Caplan M. E. Goldman M. (1981). Personal space violations as a function of height. J. Soc. Psychol. 114, 167–171.

[ref13] Carbon C. C. (2020). Wearing face masks strongly confuses counterparts in reading emotions. Front. Psychol. 11:566886. 10.3389/fpsyg.2020.566886, PMID: 33101135 PMC7545827

[ref14] Cartaud A. Ott L. Iachini T. Honoré J. Coello Y. (2020a). The influence of facial expression at perceptual threshold on electrodermal activity and social comfort distance. Psychophysiology 57:e13600. 10.1111/psyp.13600, PMID: 32437046

[ref15] Cartaud A. Quesque F. Coello Y. (2020b). Wearing a face mask against COVID-19 results in a reduction of social distancing. PLoS One 15:e0243023. 10.1371/journal.pone.0243023, PMID: 33284812 PMC7721169

[ref16] Cartaud A. Ruggiero G. Ott L. Iachini T. Coello Y. (2018). Physiological response to facial expressions in peripersonal space determines interpersonal distance in a social interaction context. Front. Psychol. 9:657. 10.3389/fpsyg.2018.00657, PMID: 29867639 PMC5949865

[ref17] Chu D. K. Akl E. A. Duda S. Solo K. Yaacoub S. Schünemann H. J. (2020). SARS-COV-2 systematic urgent review group effort (SURGE) study authors. Physical distancing, face masks, and eye protection to prevent person-to-person transmission of SARS-CoV-2 and SARS-COV-2: a systematic review and meta-analysis. Lancet 395, 1973–1987. 10.1016/S0140-6736(20)31142-9, PMID: 32497510 PMC7263814

[ref18] Coello Y. Cartaud A. (2021). The interrelation between peripersonal action space and interpersonal social space: psychophysiological evidence and clinical implications. Front. Hum. Neurosci. 15:636124. 10.3389/fnhum.2021.636124, PMID: 33732124 PMC7959827

[ref19] D’Angelo M. di Pellegrino G. Frassinetti F. (2019). The illusion of having a tall or short body differently modulates interpersonal and peripersonal space. Behav. Brain Res. 375:112146. 10.1016/j.bbr.2019.112146, PMID: 31401144

[ref20] Evans G. W. Howard R. B. (1973). Personal space. Psychol. Bull. 80, 334–344. 10.1037/h0034946, PMID: 4590526

[ref21] Gessaroli E. Santelli E. di Pellegrino G. Frassinetti F. (2013). Personal space regulation in childhood autism spectrum disorders. PLoS One 8:e74959. 10.1371/journal.pone.0074959, PMID: 24086410 PMC3781155

[ref22] Hall E. T. (1966). The Hidden Dimension. Vol. 12. New York: Doubleday & Co.

[ref23] Hayduk L. A. (1983). Personal space: where we now stand. Psychol. Bull. 94:293. 10.1037/0033-2909.94.2.293

[ref24] Hecht H. Welsch R. Viehoff J. Longo M. R. (2019). The shape of personal space. Acta Psychol. 193, 113–122. 10.1016/j.actpsy.2018.12.009, PMID: 30622020

[ref25] Iachini T. Coello Y. Frassinetti F. Ruggiero G. (2014). Body space in social interactions: a comparison of reaching and comfort distance in immersive virtual reality. PLoS One 9:e111511. 10.1371/journal.pone.0111511, PMID: 25405344 PMC4236010

[ref26] Iachini T. Coello Y. Frassinetti F. Senese V. P. Galante F. Ruggiero G. (2016). Peripersonal and interpersonal space in virtual and real environments: Effects of gender and age. J. Environ. Psychol. 45, 154–164. 10.1016/j.jenvp.2016.01.004

[ref27] Iachini T. Frassinetti F. Ruotolo F. Sbordone F. L. Ferrara A. Arioli M.. (2021). Social distance during the COVID-19 pandemic reflects perceived rather than actual risk. Int. J. Environ. Res. Public Health 18:5504. 10.3390/ijerph18115504, PMID: 34063754 PMC8196577

[ref28] Iachini T. Pagliaro S. Ruggiero G. (2015). Near or far? It depends on my impression: Moral information and spatial behavior in virtual interactions. Acta Psychol. 192, 131–136. 10.1016/j.actpsy.2015.09.003, PMID: 26386781

[ref29] Ioannou S. Morris P. Mercer H. Baker M. Gallese V. Reddy V. (2014). Proximity and gaze influences facial temperature: a thermal infrared imaging study. Front. Psychol. 5:845. 10.3389/fpsyg.2014.00845, PMID: 25136326 PMC4120854

[ref30] Johnson D. F. Druce J. D. Birch C. Grayson M. L. (2009). A quantitative assessment of the efficacy of surgical and N95 masks to filter influenza virus in patients with acute influenza infection. Clin. Infect. Dis. 49, 275–277. 10.1086/600041, PMID: 19522650

[ref31] Khosronejad A. Santoni C. Flora K. Zhang Z. Kang S. Payabvash S.. (2020). Fluid dynamics simulations show that facial masks can suppress the spread of COVID-19 in indoor environments. AIP Adv. 10:125109. 10.1063/5.0035414 [Epub ahead of print], PMID: 33173803

[ref32] Koo T. K. Li M. Y. (2016). A guideline of selecting and reporting intraclass correlation coefficients for reliability research. J. Chiropr. Med. 15, 155–163. 10.1016/j.jcm.2016.02.012, PMID: 27330520 PMC4913118

[ref33] Matuschek C. Moll F. Fangerau H. Fischer J. C. Zänker K. van Griensven M.. (2020). Face masks: benefits and risks during the COVID-19 crisis. Eur. J. Med. Res. 25, 1–8. 10.1186/s40001-020-00430-5, PMID: 32787926 PMC7422455

[ref34] Nandrino J. L. Ducro C. Iachini T. Coello Y. (2017). Perception of peripersonal and interpersonal space in patients with restrictive-type anorexia. Eur. Eat. Disord. Rev. 25, 179–187. 10.1002/erv.2506, PMID: 28260238

[ref35] Remland M. S. Jones T. S. Brinkman H. (1995). Interpersonal distance, body orientation, and touch: Effects of culture, gender, and age. J. Soc. Psychol. 135, 281–297. 10.1080/00224545.1995.9713958, PMID: 7650932

[ref36] Ruggiero G. Frassinetti F. Coello Y. Rapuano M. di Cola A. S. Iachini T. (2017). The effect of facial expressions on peripersonal and interpersonal spaces. Psychol. Res. 81, 1232–1240. 10.1007/s00426-016-0806-x, PMID: 27785567

[ref37] Ruggiero G. Rapuano M. Cartaud A. Iachini T. (2021). Defensive functions provoke similar psychophysiological reactions in reaching and comfort spaces. Sci. Rep. 11:5170. 10.1038/s41598-021-83988-2, PMID: 33664292 PMC7933359

[ref38] Ruggiero G. Rapuano M. Iachini T. (2019). Perceived temperature modulates peripersonal and interpersonal spaces differently in men and women. J. Environ. Psychol. 63, 52–59. 10.1016/j.jenvp.2019.04.004

[ref39] Sajed A. Amgain K. (2020). Corona virus disease (COVID-19) outbreak and the strategy for prevention. Eurasian J. Med. Sci. 2, 1–3.

[ref40] Santarpia J. L. Herrera V. L. Rivera D. N. Ratnesar-Shumate S. Reid S. P. Denton P. W.. (2020). The infectious nature of patient-generated SARS-CoV-2 aerosol. MedRxiv. 10.1101/2020.07.13.20041632 PMC8372686 34408261

[ref41] Schaller M. (2006). Parasites, behavioral defenses, and the social psychological mechanisms through which cultures are evoked. Psychol. Inq. 17, 96–101.

[ref42] Sicorello M. Stevanov J. Ashida H. Hecht H. (2019). Effect of gaze on personal space: a Japanese–German cross-cultural study. J. Cross-Cult. Psychol. 50, 8–21. 10.1177/0022022118798513

[ref43] Sorokowska A. Sorokowski P. Hilpert P. Cantarero K. Frackowiak T. Ahmadi K.. (2017). Preferred interpersonal distances: a global comparison. J. Cross-Cult. Psychol. 48, 577–592. 10.1177/0022022117698039

[ref44] Tajadura-Jiménez A. Pantelidou G. Rebacz P. Västfjäll D. Tsakiris M. (2011). I-space: The effects of emotional valence and source of music on interpersonal distance. PLoS One 6:e26083. 10.1371/journal.pone.0026083, PMID: 22022516 PMC3192152

[ref45] Tang S. Mao Y. Jones R. M. Tan Q. Ji J. S. Li N.. (2020). Aerosol transmission of SARS-CoV-2? Evidence, prevention and control. Environ. Int. 144:106039. 10.1016/j.envint.2020.106039, PMID: 32822927 PMC7413047

[ref46] Uzzell D. Horne N. (2006). The influence of biological sex, sexuality and gender role on interpersonal distance. Br. J. Soc. Psychol. 45, 579–597. 10.1348/014466605X58384, PMID: 16984722

[ref47] Welsch R. von Castell C. Rettenberger M. Turner D. Hecht H. Fromberger P. (2020). Sexual attraction modulates interpersonal distance and approach-avoidance movements towards virtual agents in males. PLoS One 15:e0231539. 10.1371/journal.pone.0231539, PMID: 32315317 PMC7173797

[ref48] Xiong W. Phillips M. Wang Z. Zhang Y. Cheng H. Link B. (2020). Stigma and discrimination associated with mental illness and other stigmatizing conditions in China using two cultural-sensitive measures of stigma: interpersonal distance and occupational restrictiveness. Psychol. Med., 1–10. 10.1017/s0033291720001439, PMID: 32482176

[ref49] Yang Z. (1988). An experimental study of Chinese adult space zone. Psychol. Sci. 2, 24–28.

[ref50] Yu X. Xiong W. Lee Y. C. (2020). An investigation into interpersonal and peripersonal spaces of Chinese people for different directions and genders. Front. Psychol. 11:981. 10.3389/fpsyg.2020.00981, PMID: 32581912 PMC7290242

[ref51] Zhou C. Han M. Liang Q. Hu Y. H. Kuai S. G. (2019). A social interaction field model accurately identifies static and dynamic social groupings. Nat. Hum. Behav. 3, 847–855. 10.1038/s41562-019-0618-2, PMID: 31182793

